# Comparative Study on Coffee and Spent Coffee Grounds of Arabica and Robusta Varieties of Ugandan Origin: Bioactive Properties of Extracts as Circular Resources

**DOI:** 10.3390/ijms27219906

**Published:** 2026-11-05

**Authors:** Mihaela Dinu, Auras Marius Dumitru, Ana-Maria Claudia Brezoiu, Gabriela Olimpia Isopencu, Petrica Iancu

**Affiliations:** 1 Department of Chemical and Biochemical Engineering, National University of Science and Technology POLITEHNICA Bucharest, 1, Gh. Polizu Street, Building A, Room A056, RO-011061 Bucharest, Romania; mihaela.popescu3010@upb.ro (M.D.); ana_maria.brezoiu@upb.ro (A.-M.C.B.); petrica.iancu@upb.ro (P.I.); 2 Doctoral School Chemical Engineering and Biotechnologies, National University of Science and Technology POLITEHNICA Bucharest, RO-011061 Bucharest, Romania; auras.dumitru@upb.ro

**Keywords:** Arabica and Robusta varieties of Ugandan origin, roasted and spent coffee grounds, polar and non-polar solvents, polyphenolic and lipid fractions, antioxidants, antimicrobials

## Abstract

Coffee consumption is increasing yearly, and so is the quantity of spent coffee grounds, a by-product that still contains bioactive compounds. The goal of this work is to evaluate the lipid fractions (LFs) and polyphenolic fractions (PFs) from roasted coffee grounds (RCG; two varieties of Ugandan origin: Arabica and Robusta) and from the corresponding spent coffee grounds (SCG) extracted with solvents of opposite polarities. A qualitative and quantitative analysis of the extracts was performed using GC-FID, ^1^H-NMR, Folin–Ciocalteu, HPLC-PDA, DPPH and antibacterial activity methods. A high-quality LF, coffee oil (91.82 g triacylglycerides/100 g LF, 49.85 mol% saturated fatty acids and 50.15 mol% unsaturated fatty acid chains, 8.98 mg GAE/g LF, 20.91% DPPH inhibition), with high extraction yield (10.34 g/100 g SCG), can be recovered from SCG of the Ugandan Arabica variety. In addition, SCG of both Ugandan Arabica and Robusta varieties are promising resources for the recovery of high-quality PF (12.30 and 7.85 mg GAE/g PF, 0.684 and 0.837 mg/g PF chlorogenic acids, 85.17% and 73.95% DPPH inhibition) with good extraction yields (9.35 and 5.63 g/100 g SCG) and with antimicrobial activities that remain after the coffee brewing step. These results highlight SCG of Ugandan origin as a promising resource of bioactive compounds for further valorization.

## 1. Introduction

Coffee ranks first among the most consumed beverages in the world and second among the most traded commodities, surpassed only by crude oil [1]. Coffee consumption is steadily increasing year by year, and with it, the amount of spent coffee grounds as a solid by-product of the coffee brewing process is also rising. Processing one ton of raw green coffee beans generates 650 kg of spent coffee grounds as a by-product, with the annual global production reaching 6–8 million tons [2,3,4]. This by-product has a high moisture content (50–65%) and is considered waste, although it represents a renewable source containing important bioactive compounds (that remain after brewing coffee) with potential for valorization [5,6]. Spent coffee grounds represent an underexplored material at the industrial level, mainly in terms of non-polar compounds, because the coffee brewing method removes mostly water-soluble compounds [7,8]. Spent coffee grounds contain 35–57% carbohydrates, 19–33.6% lignin, 2–25% lipids, 4–17.5% proteins, 1–3% phenolic compounds, 2–9% dietary fiber and 0.7–4% minerals [9,10]. Coffee oil contains fatty acids, sterols and tocopherols as major components and polyphenolic compounds as minor components. The distinct fatty acid profile of coffee oil is characterized by high levels of linoleic C18:2 (36–42%) and palmitic C16:0 (32%) acids and low levels of oleic C18:1 (10–12%) and stearic C18:0 (7–10%) acids, is rich in both polyunsaturated acids (PUFAs) and saturated fatty acids (SFAs) and differs from that of common edible vegetable oils [2,11,12,13]. Coffee oil exhibits a PUFA/SFA ratio > 1, which confers significant health benefits and makes it suitable for use in nutraceuticals and dietary supplements [14]. In addition, the tocopherol levels are significantly higher than in most commercial oils [7]. In terms of polyphenolics, spent coffee grounds extracts present significant amounts of phenolic acids (chlorogenic acid, caffeic acid, ferulic acid, quinic acid, tannic acid, ellagic acid), flavonoids (quercetin, catechin, epicatechin, rutin, kaempferol), diterpenes (cafestol, kahweol), melanoidins and minor amounts of alkaloids (caffeine, trigonelline) which possess important biological properties, including antioxidant, anti-inflammatory, antiallergic, antimicrobial, anti-aging, anticancer and antiglycation activities [3,11,15,16]. The bioactive profiles of spent coffee grounds lipid and polyphenolic fraction extracts are equal to or even higher than those of the coffee beverage, making this by-product functionally competitive [14,17].

The composition of spent coffee grounds differs depending on variety, geographical origin, processing conditions such as roasting degree (light, medium, dark), grinding size or coffee brewing methods [8,11,12]. Under these conditions, spent coffee grounds are usually incinerated, used as low-value fertilizers [2] or discarded in landfills, leading to negative environmental impacts due to uncontrolled decomposition and the generation of greenhouse gases such as methane and carbon dioxide [5], as well as the presence of some bioactive compounds (caffeine, chlorogenic acid) that can be toxic to water and soil [3,14]. Recent studies highlight the increasing interest in spent coffee grounds valorization due to their residual content of bioactive compounds, including polyphenols, lipids, proteins, and minerals. Spent coffee grounds represent a promising functional ingredient for sustainable applications in food science, cosmetics, biofuels and biodegradable packaging [18].

Among commercially important coffee species, Arabica (*Coffea arabica*) and Robusta (*Coffea canephora*) differ in chemical composition and sensory profile. Arabica coffee is generally considered superior in quality because it possesses a milder and more aromatic flavor, while Robusta is characterized by a stronger taste profile with increased bitterness and astringency [19]. The lipid fraction in Arabica coffee is around 17%, while the lipid content in Robusta coffee is usually lower, up to 10% [13]. However, Robusta coffee tends to contain higher levels of chlorogenic acids, caffeine, and total phenolic compounds compared to the Arabica variety [20].

Depending on the origin, coffee from Uganda is reported to have the highest concentrations of chlorogenic acids in both coffee varieties and of saturated fatty acid chains [21]. Uganda is the second-largest coffee producer in Africa and the sixth-largest globally and is unique among Africa’s coffee-producing countries, cultivating both Arabica and Robusta coffee. Arabica coffee production accounts for 23% and is localized in high-altitude areas, which have cooler temperatures and well-drained soils, while Robusta coffee accounts for 77% and is localized in low-altitude regions [22]. Relevant studies show the Ugandan coffee beans antioxidant and antidiabetic properties due to their unique phytochemical profiles [21,23], because native Robusta coffee is rich in caffeine, chlorogenic acids, and free amino acids, while Arabica coffee contains lipids, sugars, and volatile aromatic compounds [24,25].

The present work proposes to investigate the valorization potential of using and exploiting a by-product from the food industry, namely spent coffee grounds of known origin, by comparatively analyzing the amounts of bioactive compounds from two coffee varieties, Arabica and Robusta of Ugandan origin. Freshly roasted coffee grounds of known origin and their spent coffee grounds obtained by a water-pulsed extraction process are used for extraction with polar and non-polar solvents. Most studies used coffee blends or spent coffee grounds without controlled origin, and very few studies have considered the behavior of solvents with different polarities for the extraction of bioactive compounds from coffee and from the spent coffee grounds resulting from it. In summary, this study investigates the potential of spent coffee grounds valorization compared to roasted coffee grounds from the two native varieties from Uganda (Arabica and Robusta) through extraction with different organic solvents (hexane and ethanol). The aim is to evaluate the effects of solvent polarity, coffee matrix type and coffee variety on the extraction yield of bioactive compounds and to compare their extraction efficiency in terms of the compositions of polyphenolic and lipid fractions and their effects. The analytical characterization of the extracts includes the determination of total phenolic contents, polyphenolic and lipid fractions compositions, antioxidant activity, antimicrobial activity, enabling a comparative evaluation between roasted and spent coffee grounds from Arabica and Robusta varieties of Ugandan origin. The controlled origin of the spent coffee grounds used for the extraction of bioactive compounds ensures a stable concentration of these extracts, as well as clear information for those interested in utilizing them within the cosmetic and food industries.

## 2. Results

### 2.1. Extraction of Lipid and Polyphenolic Fractions from Coffee Samples

The Soxhlet extraction method was used to isolate the lipid and the polyphenolic fractions from two different coffee matrix types, roasted coffee grounds (RCG) and spent coffee grounds (SCG), from both Arabica and Robusta varieties of Ugandan origin. Hexane was used as a non-polar solvent to isolate the lipid fractions (LFs), while ethanol was used as a polar solvent to recover the polyphenolic fractions (PFs). RCG and SCG samples (green coffee beans, roasted coffee beans and roasted coffee grounds and spent coffee grounds) and extracts are illustrated in Figure 1a for the Arabica variety and in Figure 1b for the Robusta variety to highlight the changes in coffee beans matrix types and color between raw materials and processed materials for both varieties. In addition, the color of the extracts resulted from processed coffee beans from both varieties is highlighted. All the RCG and SCG extracts differ in color due to the types of bioactive compounds and the degree of roasting. Arabica and Robusta green coffee beans were roasted, and the roasting degree values of the RCG samples were 105 and 103, respectively, indicating a medium-light roast profile, according to the Tonino Scale (100–115). In addition, the extracts’ consistencies are different: the LFs isolated with hexane are oily, while the PFs are hydrophilic.

Regarding the extraction efficiency using the Soxhlet method, extraction yields are evaluated, and the obtained mean values are presented in Table 1. The extraction yields from RCG and SCG coffee samples vary between 4.09 and 12.74 g extract/100 g coffee sample depending on: coffee variety, coffee matrix type and extraction solvents.

Regarding the influence of the coffee variety, differences in extraction yields were observed, with Arabica showing higher values than Robusta, for both RCG (>75% for LFs and >36% for PFs) and SCG (>150% for LFs and >66% for PFs), in accordance with the values reported by other studies [2,13,26].

SCG extraction yields compared with those of RCG are significantly lower for Robusta—both in terms of LF (as Robusta contains fewer oils) and PF (as Robusta contains more soluble solids that were separated during the pressurized water extraction process): 10.34 g LF/100 g of Arabica SCG vs. 4.09 g LF/100 g of Robusta SCG or 9.35 g PF/100 g of Arabica SCG vs. 5.63 g PF/100 g of Robusta SCG.

The extraction solvents influence the type of fraction that can be isolated. Hexane—a non-polar solvent that exhibits an affinity for lipid molecules (which are also non-polar) and ethanol—a polar solvent containing -OH groups that allow it to form hydrogen bonds—were used as extraction solvents. The obtained extraction yields demonstrate the influence of the polarity of the solvent used on the extraction yields. A notable difference is observed in the extraction yields for the Robusta variety, for both RCG and SCG: 6.31 g LF/100 g RCG vs. 9.30 g PF/100 g RCG and 4.09 g LF/100 g SCG vs. 5.63 g PF/100 g SCG. These values are comparable with extraction yields reported by other studies when hexane is used as an extraction solvent for RCG (12–20 g LF/100 g RCG [13]) and for SCG (8.82–16.7 g LF/100 g SCG [6,26,27,28,29,30,31,32,33,34]). Extraction yields obtained using ethanol show an increase of 30–40% for Robusta and 10–11% for Arabica (with a small difference for SCG), compared with those obtained using hexane. Ethanol extraction yield values showing the same trend were observed in other studies, with values of 3.18–15.95 g PF/100 g SCG [26,28,29,30,32].

### 2.2. Glycerides Composition of Lipid Fractions

Gas chromatography coupled with the flame ionization detection method (GC-FID) was used to identify the acylglycerides present in LF extracted from RCG and SCG coffee matrix types from both Arabica and Robusta varieties of Ugandan origin (Figure S1—Supplementary Material). The main compounds (Table 2) are triacylglycerides (91.82–92.41 g/100 g LF), diacylglycerides (3.08–3.15 g/100 g LF), very small quantities of monoacylglycerides and 4.21–4.80 g/100 g of LF free fatty acids, which contribute to the oil’s acidity (coffee oil acidity index values range from 3 to 19 [35]). Other triacylglyceride values of 81.16–96.25 g/100 g oil [27,30] were reported from LF extracted with hexane from SCG samples. All the LF samples presented similar proportions of triacylglycerides because these compounds are not extracted with water during the pulsed extraction process involved in the preparation of coffee beverages [6]. A relevant observation was presented by Zeng et al. [2], who claimed that the types of fatty acid chains linked to the glycerol molecule influences the physical and chemical characteristics of the coffee oil; therefore, it is necessary to identify compounds and their proportions in LFs.

### 2.3. Proton Nuclear Magnetic Resonance (^1^H-NMR) Characterization and Fatty-Acyl Chains Composition of Lipid Fractions

The ^1^H-NMR spectra of LF exhibited the characteristic resonances of fatty-acyl chains and esterified glycerol groups (Figure S2, Tables S1 and S2—Supplementary Material). Following the assignments reported by Knothe and Kenar [36], signal B at approximately 0.85 ppm corresponds to the terminal methyl groups of most fatty-acyl chains, while the intense resonance C at 1.24 ppm originates from internal methylene groups. Signals D and F, at approximately 1.64 and 2.26 ppm, are assigned to methylene protons in the β- and α-positions relative to the ester carbonyl, respectively. The resonances at 2.02 ppm (E), 2.76 ppm (G), and 5.29 ppm (J) correspond to allylic, bis-allylic, and olefinic protons, respectively. The glycerol-backbone signals occur around 4.19 ppm (H, *sn*-1/*sn*-3 methylene protons) and 5.15 ppm (I, *sn*-2 methine proton). These assignments provide the basis for estimating fatty-acyl class composition using the computational approach of Chira et al. [37].

LF from Ugandan coffee samples extracted with hexane contained substantial proportions of saturated and di-unsaturated fatty-acyl chains, accounting for 46.96–52.74 mol% and 31.96–44.14 mol%, respectively (Table 3). Mono-unsaturated chains contributed 8.90–15.64 mol%. The di-unsaturated fraction, predominantly attributable to linoleate, decreased across the sample sequence from A-RCG-H to R-SCG-H (higher for RCG), whereas the saturated fraction generally increased (higher for SCG). The mono-unsaturated contribution, mainly represented by oleate, increased from 8.90 mol% in A-RCG-H to 15.64 mol% in R-RCG-H and remained similar in R-SCG-H (15.31 mol%). Thus, A-RCG-H exhibited the highest di-unsaturated contribution, while R-SCG-H showed the highest saturated contribution.

The overall composition agrees with the linoleic–palmitic profile characteristic of coffee bean oils. Dong et al. [40] reported 40.67–43.77% linoleic acid, 36.57–38.71% palmitic acid, 8.54–9.94% oleic acid and 6.70–7.11% stearic acid in green Arabica coffee oils obtained by different extraction methods. Their total saturated-fatty acid fraction (46.65–48.85%) is close to the values obtained for A-RCG-H, A-SCG-H and R-RCG-H, whereas R-SCG-H exhibited a somewhat higher saturated contribution. A-RCG-H also showed a di-unsaturated fraction close to their reported linoleic-acid range; R-RCG-H and R-SCG-H instead displayed lower di-unsaturated and higher mono-unsaturated contributions. A higher proportion of di-unsaturated fatty acids in the Arabica coffee variety than in Robusta was also observed by Zeng et al. [2], who analyzed the oil composition of several coffee samples from both varieties and at different roasting levels. However, it can be observed that the di-unsaturated fatty acid content of LF from Arabica SCG is even higher than that of Robusta RCG. A very weak resonance at approximately 0.96 ppm was observed in A-RCG-H and A-SCG-H (Figure S2—Supplementary Material), consistent with a minor contribution from n-3 fatty-acyl chains, most plausibly α-linolenate (C18:3). Its low intensity did not permit reliable integration under the acquisition conditions employed; consequently, it was interpreted qualitatively rather than assigned a precise concentration. No corresponding resolved resonance was detected in R-RCG-H or R-SCG-H, although non-detection does not establish complete absence of C18:3. The assignment also requires consideration of spectral specificity: Tociu et al. [41] emphasized that the terminal methyl groups of n-3 fatty-acyl chains and esterified butyryl moieties can overlap near 0.96 ppm. Since butyryl moieties are characteristic constituents of dairy triacylglycerols [42], an n-3 contribution is more plausible in a coffee-derived oil.

The degree of unsaturation of the acyl chains from LF was also evaluated using the iodine value and the saponification value. The iodine values of LF ranged from 74.03 to 77.07 g I_2_/100 g LF. The highest value occurred in A-RCG-H, consistent with its greater di-unsaturated contribution, while R-RCG-H and R-SCG-H exhibited similar values despite differences in their fatty-acyl class distributions. The values were lower than those reported by Raba et al. [43] for oils extracted from green and roasted Arabica beans, 88.91 ± 2.47 and 86.24 ± 1.88 g I_2_/100 g oil, respectively, but higher than the 51.89–60.21 g I_2_/100 g range reported by Dong et al. [40]. This variation highlights the importance of sample characteristics, extraction conditions and analytical approach when comparing coffee oil quality indices.

The calculated saponification values of LF ranged from 202.81 to 208.39 mg KOH/g LF. Ivanova et al. [39] established the relationship between NMR-derived saponification value and average triacylglycerol molecular mass: shorter average fatty-acyl chains generally produce higher values. Within a predominantly C16–C18 system, a greater contribution from palmitate rather than stearate would therefore increase the saponification value. Although the present ^1^H-NMR-based approach for computing the saturated-fatty-acyl fraction does not independently resolve C16:0 and C18:0, this interpretation is qualitatively consistent with the predominance of palmitic over stearic acid reported for coffee oils by Dong et al. [40]. The values obtained here exceeded those reported by Raba et al. [43] for green and roasted coffee oils, 185.86 ± 3.10 and 179.29 ± 3.63 mg KOH/g, respectively, but fell within the broader 88.49–230.04 mg KOH/g range reported by Dong et al. [40] across extraction methods. Such agreement in range should be interpreted cautiously because coffee oil is a complex lipid extract, whereas the ^1^H-NMR calculation relies on structural assumptions concerning the saponifiable lipid fraction. Overall, the spectra and calculated indices support a coffee oil profile rich in saturated and linoleate-type chains, accompanied by smaller mono-unsaturated contributions and, in A-RCG-H and A-SCG-H, a weak feature consistent with minor n-3 constituents (C18:3).

### 2.4. Total Phenolic Contents (TPC) of Lipid and Polyphenolic Fractions

To analyze the TPC of LF and PF of Ugandan coffee samples, the results were expressed as mg of reference phenolic compound (RPCE)/mg extract using four reference standards (gallic acid, caffeic acid, ferulic acid and tannic acid), which have different chemical structures and varying numbers of hydroxyl (–OH) groups, which means that they will react with different intensities. The TPC values obtained with all four standards (Figure 2a,b) show the same trend: higher concentrations in RCG and lower concentrations in SCG, with higher values observed for ferulic and caffeic acids—compounds whose structures are similar to those identified in the analyzed samples.

For comparison with other studies, the results are expressed in mg of gallic acid equivalents (GAE)/g of extract. As it can also be seen in Figure 2a,b, higher TPC values of 13.86–14.23 mg GAE/g RCG ethanolic extracts and 7.85–12.30 mg GAE/g SCG ethanolic extracts from both varieties are found mostly in isolated PFs, due to the polarity of ethanol, which favors the solubilization of phenolic compounds.

Comparable values of TPC for PFs extracted from SCG with ethanol were reported in other studies: 3.52 mg GAE/g [17], 5.45–9.43 mg GAE/g [44], 13.12 mg GAE/g [32] and 15.99 mg GAE/g [45]. For Ugandan coffee extracts, the highest TPC values of 12.65–14.23 mg GAE/g was obtained from the LF and PF belonging to Arabica variety. This trend was also observed by Hasbullah et al. [46], who reported that Arabica extracts exhibit superior values compared with Robusta samples. Other studies focused on SCG reported significantly lower values in LF extracted from SCG with hexane as 0.05–0.21 mg GAE/g recovered from both Arabica and Robusta varieties [2], 0.73 mg GAE/g [32] and 0.94 mg GAE/g [16].

### 2.5. High-Performance Liquid Chromatography with Photodiode Array Detection (HPLC-PDA) Analysis of Polyphenolic Fractions

Chromatograms of PF prepared from RCG and SCG samples from Arabica and Robusta varieties of Ugandan origin are presented in Figure S3 (Supplementary Material). From the twenty-seven available standard substances, up to eleven compounds were identified in all the ethanolic extracts. The identified compounds are from three different classes: hydroxycinnamic acids (neochlorogenic, caftaric, chlorogenic, caffeic, p-coumaric, trans-ferulic and chicoric acids), flavonoids ((-) epicatechin and quercetin) and phenolic acids (gallic and protocatechuic acids). The RCG extracts presented larger amounts of polyphenols than their corresponding SCG extracts (Table 4), regardless of coffee variety, in agreement with the TPC results presented above. The amount of polyphenols that was found in the extracts varied depending on the coffee variety and matrix type.

In all the analyzed extracts, the following polyphenols were identified with the first value corresponding to RCG and the second to SCG, respectively: chlorogenic acid (0.776–2.210 mg/g PF and 0.514–0.519 mg/g PF), neochlorogenic acid (0.386–0.971 mg/g PF and 0.170–0.318 mg/g PF) and lower amounts of caftaric acid (0.040–0.058 mg/g PF and 0.011–0.050 mg/g PF), caffeic acid (0.048–0.064 mg/g PF and 0.013–0.041 mg/g PF) and chicoric acid (0.150–0.358 mg/g PF and 0.080–0.144 mg/g PF). Moreover, gallic acid and epicatechin were only found in the A-RCG-E extract (0.022 ± 0.003 mg/g and 0.024 ± 0.000 mg/g), p-coumaric acid only in the A-RCG-E and A-SCG-E extracts in low amounts (0.006–0.009 mg/g PF), and quercetin was only found in A-SCG-E (0.008 ± 0.000 mg/g PF). Protocatechuic acid was identified in all the ethanolic extracts except for the R-SCG-E extract in the range of 0.010–0.025 mg/g PF. Due to the unavailability of caffeine standard substance, we were unable to quantify it in the prepared extracts. However, caffeine in ethanolic extracts probably corresponds to the peak at 14.4 min, as the UV-spectra is similar to the available literature data and elutes before caffeic acid, in agreement with other literature data [47,48].

Several studies regarding the PF content in RCG have been reported in the literature. A study presented several polyphenolic compounds found in coffee samples from different varieties (Robusta, Arabica or blends), including one from Uganda. In the RCG water extracts, they reported the identification of compounds similar to those presented in this study, including gallic, chlorogenic, ferulic, p-coumaric and caffeic acids, using the HPLC-HR MS technique [49]. Another study presented the composition of a macerated RCG extract from Robusta (Uganda), which contained significantly lower amounts of compounds, probably due to the polar nature of the extraction solvent and low extraction temperature (room temperature), as follows: chlorogenic acid 6.62 mg/100 g, caffeic acid 0.04 mg/100 g and protocatechuic acid 0.14 mg/100 g, respectively [23]. Another study focused on Brazilian RCG extracts prepared in boiling water with different roasting treatments of either conventional or organic origin in fresh form or after storage. Their extracts presented gallic (0.38–1.45 mg/g), chlorogenic (0.02–6.00 mg/g), caffeic (0.058–0.15 mg/g) acids and quercetin (0.02–0.11), as well as caffeine, quercetin and kaempferol derivatives and salicylic acid. This study showed several reductions in the amounts of polyphenolic compounds during storage, emphasizing that fresh, lightly roasted coffee contained the highest amount of phytocompounds [50]. In addition, hydroethanolic extracts from green coffee beans or enzymatically assisted extracts showed significantly lower amounts of polyphenols than those presented in this study, with chlorogenic acid/100 g being found in the largest amount (4.69–17.7 mg), probably due to the absence of a roasting process which could enrich secondary metabolite formation [51].

Concerning the literature analysis of PFs from SCG, a study on hydroethanolic SCG from a blend of 90% Arabica and 10% Robusta using either pressurized liquid extraction or magnetic stirring emphasized the identification of several similar compounds compared to our study: gallic acid (0.09–0.12 mg/g), neochlorogenic acid (0.14–0.25 mg/g), chlorogenic acid (1.37–1.53 mg/g), caffeic acid (0.62 mg/g), p-coumaric acid (0.40–0.44 mg/g), (-) epicatechin (2.27–2.94 mg/g) and quercetin (0.83–1.43 mg/g). The authors also mentioned different amounts of syringic, ferulic acids, as well as catechin hydrate and rutin, which were not detected in our extracts. However, in comparison with our results, larger amounts of flavonoids were identified, but a smaller amount of hydroxycinnamic acid polyphenols [45]. Andrade et al. [47] prepared extracts in μ-QuenChers and a mixture of acetonitrile/ethyl acetate = 1/1 (v./v.) from SCG from Guatemala, Colombia, Brazil, Timor and Ethiopia and identified neochlorogenic (8.3–104 mg/100 g DW), chlorogenic (167.3–385.3 mg/100 g DW) and caffeic acids (3.7 mg/100 g DW). The reported amounts of polyphenols are higher than those reported in this study ranging from 0.95 to 2.96 mg/100 g DW, 2.89 to 4.85 mg/100 g DW and 0.07 to 0.38 mg/100 g DW for neochlorogenic, chlorogenic and caffeic acids, respectively.

### 2.6. Antioxidant Activities of Lipid and Polyphenolic Fractions

The antioxidant activities of LF and PF from RCG and SCG samples belonging to Arabica and Robusta varieties of Ugandan origin, assessed by the DPPH (2,2-diphenyl-1-picrylhydrazyl) free radical-scavenging assay, are illustrated in Figure 3.

DPPH inhibition values were calculated using Equation (1) (Section 4.8).

For both Arabica and Robusta varieties, PFs vs. LFs from RCG present higher values of DPPH ratios (2:1 and 5:1). For SCG extracts, LFs present higher values of DPPH ratios as 4:1 for both varieties. These results are below those reported by Romano et al. [32] for SCG originating from a blend of Arabica and Robusta, for a DPPH ratio of 9:1.

As it can be seen, the antioxidant activity is presented mostly for the ethanolic extracts, regardless of the type of coffee variety or sample due to the polarity of the solvent that enables the recovery of polar phenolic compounds. Additionally, non-polar extracts activities are linked to the fatty acids and tocopherols [52]. The antioxidant activity is in agreement with the TPC results presented above.

### 2.7. Antimicrobial Activities of Lipid and Polyphenolic Fractions

The antimicrobial activity was assessed by analyzing the response of two microorganisms: a Gram-negative bacterium, *Escherichia coli* (*E. coli*), and a Gram-positive bacterium, *Bacillus subtilis spizizenii Nakamura* (*B. subtilis*). Coffee extracts applied to sterile discs produced a total inhibition zone in mm measured with Quatros 1–150 mm caliper, which was evaluated in triplicate experiments. The control samples consisted of the solvent used for extraction and an inert compound, water, respectively, and their effects were eliminated when determining the effective zone of inhibition. The results regarding antimicrobial activity for RCG and SCG extracts, expressed as the zone of inhibition, calculated by subtracting the disc diameter and the solvent effect and dividing by 2 to obtain IZ (mm) as a radius. The obtained IZ values are presented in Figure 4, and the samples in Petri dishes are illustrated in Figure S4 (Supplementary Material).

For RCG extracts (Figure 4a), PFs from the Arabica variety exhibited noticeable anti-microbial activity, whereas PFs from the Robusta variety showed significant antimicrobial activity only against a Gram-positive bacterium. LF of RCG and SCG extracts from both varieties displayed weak antimicrobial activity against *E. coli* but considerably more pronounced activity against *B. subtilis*, particularly the fraction derived from the Arabica variety. In the case of SCG (Figure 4b), the results indicate noticeable antimicrobial activity against *B. subtilis* for both PFs and LFs—particularly with the Arabica variety, which yielded slightly higher IZ values than the Robusta variety. Antimicrobial activity against *E. coli* was low for both SCG Arabica fractions; however, PFs from the Robusta variety demonstrated significant antimicrobial activity against this Gram-negative bacterium. The antibacterial activity of PFs is consistent with studies in the literature, with effective inhibition zone values not exceeding 5 mm [53,54]. Additionally, the antibacterial effect of SCG extracts is also evident—a topic that remains a subject of ongoing debate [15,55]. The results indicate antimicrobial activity in the fractions derived from SCG, suggesting that this waste material is suitable for further valorization.

## 3. Discussion

The obtained results are in line with the proposed goal of this work, namely to determine superior quality extracts if SCG from RCG with known origin are used. To conduct a detailed analysis of the factors determining variations in the compounds recovered from SCG, the most widely grown and consumed coffee varieties worldwide—Arabica and Robusta—were used. Ugandan coffee varieties were selected due to their content of lipids, sugars and volatile aromatic compounds (Arabica) and due to their content of caffeine, chlorogenic acids, and free amino acids (Robusta), these special contents being influenced by geographical position and climate conditions. Their SCG are used to add value to this waste material by producing extracts rich in chlorogenic acids and triacylglycerides, for further use in the cosmetic, pharmaceutical or food industries.

For the recovery and analysis of the bioactive compounds, the Soxhlet extraction method using two solvents with opposite polarities, ethanol and hexane, to obtain both polar and non-polar compounds was applied. The coffee used to produce SCG was also analyzed and considered as a reference to determine the percentage of compounds of interest lost when this waste is discarded. Furthermore, the processing methods of the green coffee beans—such as coffee roasting, roasted coffee grinding, roasted coffee grounds brewing and spent coffee grounds drying—were kept consistent.

The *quantitative analysis* performed through extraction experiments shows the different extraction yields depending on the RCG variety (11.09–12.74 g/100 g Arabica samples vs. 6.31–9.30 g/100 g Robusta samples), coffee matrix type (6.31–12.74 g/100 g RCG vs. 4.09–10.34 g/100 g SCG) and the polarity of extraction solvents (10.34–11.09 g/100 g Arabica samples using hexane and 5.63–9.30 g/100 g Robusta samples using ethanol). The extraction yields of Arabica samples are higher than those of Robusta for both PFs and LFs because Robusta contains more soluble compounds extracted during pressurized brewing. The waste nature of SCG and their significant amounts of LFs and PFs highlight the potential of the residues from Ugandan Arabica variety to be considered a promising source for recovering important quantities of coffee oil and polyphenolic extracts.

The *qualitative analysis* of the extracts from Ugandan SCG Arabica variety shows that this residue presents significant quality in terms of lipid and phenolic contents and antioxidant and antimicrobial activities, after pressurized brewing.

*Lipid fraction analysis*: The quality of these extracts was evaluated by GC-FID, ^1^H-NMR, Folin–Ciocalteu, DPPH and antimicrobial disk diffusion methods. Both the LFs extracted from Ugandan Arabica and those extracted from the Robusta samples present high composition in acylglycerides, mainly in triacylglycerides (>90 g/100 g LF). Smaller percents of di- and mono- acylglycerides were identified and free fatty acids (4.21–4.80 g/100 g LF). By ^1^H-NMR characterization of LF, substantial proportions of saturated (46.96–52.74 mol%, mainly C16:0 + C18:0) and di-unsaturated fatty-acyl chains (31.94–44.14 mol%, mainly C18:2) and smaller mono-unsaturated chains (8.90–15.64 mol%, C18:1) were identified. These results are in agreement with the evaluated iodine values, which ranged from 74.03 to 77.07 g I_2_/100 g LF and with values reported in other works. Consequently, based on these results, LFs extracted from Ugandan coffee varieties support a coffee oil profile rich in saturated and linoleate-type chains, accompanied by smaller mono-unsaturated contributions. The LFs belonging to the Arabica variety have higher amounts of unsaturated fatty acids compared with the Robusta samples, as these compounds are known to be responsible for the antioxidant capacity of coffee oil. Moreover, it was observed that LF from Arabica SCG has higher amounts of unsaturated fatty acids even compared to the Robusta RCG LFs, making this by-product valuable and non-disposable. The Robusta variety shows a slightly higher trend toward fatty acid saturation and higher percentages of oleic acid compared to Arabica. Due to this balanced percentage between saturated and unsaturated chains, these lipid fractions extracted from Ugandan coffee variety should be promising sources of lipids for industries.

Since the compositions of LFs isolated from RCG and SCG using hexane are comparable, because the preparation of coffee targets the hydrophilic polyphenols, it would be expected that their activities to be closer. However, as observed, there are some differences between them in the TPC of these extracts (8.98–12.65 mg GAE/g LF for Arabica extracts and 0.85–4.61 mg GAE/g LF for Robusta extracts). The phenolic compounds are concentrated in PF isolated with ethanol; thus, the presence of phenolics in LF should be reduced. Nevertheless, the bioactive lipid compounds such as fatty acids and tocopherols can protect the phenolic compounds against degradation during extraction. The Arabica variety is known to contain higher lipids than Robusta, as was also determined in the current study. Additionally, thermal degradation of phenolic compounds can occur during coffee preparation. A high lipid content enables the Arabica variety to better protect phenolic compounds than the Robusta variety against degradation. The Ugandan Arabica variety LFs present values of DPPH inhibition between 20.91 and 34.63%, due to the content of unsaturated fatty acid chains and tocopherols, which present antioxidant activity, which is in agreement with the results obtained for TPC.

LFs from RCG and SCG extracts from both varieties displayed weak antimicrobial activity against *E. coli*, but considerably more pronounced activity against *B. subtilis*, particularly the fraction derived from the Arabica variety. Regarding the Gram-positive bacterium *B. subtilis*, the two analyzed fractions (RCG-H-BS and SCG-H-BS) show a specific antibacterial activity: the variation in IZ recorded for LF from the Arabica variety is very small (IZ = 2.5 mm for RCG-H-BS and IZ = 2.67 mm for SCG-H-BS), while for the Robusta variety, LF exhibits higher antimicrobial activity when derived from SCG (IZ = 2.00 mm for SCG-H-BS) compared to the fraction derived from RCG (IZ = 0.67 mm for RCG-H-BS). LF from Arabica SCG (A-SCG-H) exhibited a higher iodine value than the corresponding fraction from Robusta (R-SCG-H). This indicates a greater abundance of polyunsaturated fatty acids, which possess a superior ability to penetrate bacterial cell envelopes. Gram-positive bacteria are the most susceptible to these compounds, as they lack the protective outer lipopolysaccharide membrane found in Gram-negative bacteria. In the SCG of both varieties, the presence of polyunsaturated fatty acids—as evidenced by similar iodine values—also manifests as significantly higher antimicrobial activity.

*Polyphenolic fraction analysis*: The quality of these extracts was evaluated by HPLC-PDA, Folin–Ciocalteu, DPPH and antimicrobial disk diffusion methods. The PF recovered from SCG is concentrated in polar polyphenolic bioactive compounds that possess important health activities, comparable to RCG extracts. Three different classes of compounds, namely, hydroxycinnamic acids (neochlorogenic, caftaric, chlorogenic, caffeic, p-coumaric, trans-ferulic and chicoric acids), flavonoids ((-) epicatechin and quercetin) and phenolic acids (gallic and protocatechuic acids) were identified in ethanolic extracts from Ugandan coffee samples by HPLC-PDA analysis. Of these, higher amounts were found in RCG vs. SCG extracts. Although chlorogenic and neochlorogenic acids are predominantly in all extracts, with higher values of 1.162–3.181 mg/g PF in RCG, significant amounts still exist in SCG extracts (0.684–0.837 mg/g PF) for both coffee varieties, which show the antioxidant nature of these extracts.

The TPC in PF of SCG of the Arabica variety represents 12.30 mg GAE/g PF from RCG, and 7.85 mg GAE/g PF for the Robusta variety, respectively. For RCG extracts, it was observed that TPC is similar for both Arabica and Robusta varieties. For the PF of RCG and SCG from both varieties, a similar behavior can be observed. RCG-E extracts contain a higher phenolic contents than SCG-E extracts, because a portion of phenolic compounds is removed during the coffee preparation stage. In addition, A-SCG-E presents a higher phenolic content than R-SCG-E. Regarding the antioxidant activity, PF isolated from SCG of the Arabica variety has higher activity compared to the Robusta variety (85.17% DPPH inhibition vs. 73.95% DPPH inhibition). For the Robusta variety, the antioxidant activity of SCG is 86% of that of the RCG sample. In addition, the Arabica variety exhibited higher activities and greater amounts of bioactive compounds than the Robusta variety in PF extracts.

Regarding the antimicrobial activity of the fractions obtained via extraction from the Arabica variety, significant differences against *E. coli* are observed for the PF fraction derived from RCG, which is substantially higher (approximately four times greater) than the corresponding fraction from SCG. The results obtained for Gram-negative bacterium *E. coli* using the PF from RCG of the two analyzed coffee varieties (A-RCG-E-EC, IZ = 4.40 mm and R-RCG-E-EC, IZ = 0.33 mm) are contradictory. HPLC-PDA analysis revealed high levels of chlorogenic acid in both samples, a compound with potential antimicrobial effects, as noted in the literature [56]. The experimental data indicates that the effect of chlorogenic acid is shielded by the presence of other compounds present in the RCG ethanolic extract from the Robusta variety. Since the Robusta variety contains more caffeine [21], there is greater competition for membrane permeability in *E. coli*; consequently, the antimicrobial activity of the PF from Robusta RCG is significantly lower than that of the Arabica variety. A Gram-positive bacterium (such as *B. subtilis*) exhibits greater sensitivity to chlorogenic acid. This is due to the absence of a protective outer lipopolysaccharide membrane (which is present in Gram-negative bacteria). Because of its hydrophilic nature, chlorogenic acid can more easily traverse the porous structure of the peptidoglycan layer within the bacterial cell wall. PF from SCG demonstrated significant antibacterial activity against *E. coli* in the Robusta variety (R-SCG-E-EC, IZ = 4.92 mm). This is because, following the extraction of caffeine during coffee brewing [57], the remaining SCG contain a sufficient concentration of polyphenols to inhibit bacterial growth, a result not observed at the Arabica variety.

## 4. Materials and Methods

### 4.1. Chemicals and Standards

Ethanol and hexane were of analytical grade and purchased from Sigma Aldrich (Munich, Germany) and were used for extraction experiments. n-Heptane (99% purity) and hexane from Sigma Aldrich (Munich, Germany) were used for the GC-FID analysis. CDCl_3_ from Sigma Aldrich (Munich, Germany) was used for ^1^H-NMR analysis. Ethanol, DPPH, 6-hydroxy-2,5,7,8 tetramethylbroman-2-carboxylic acid (Trolox), Folin–Ciocalteu reagent (2 N), gallic acid, caffeic acid, ferulic acid, tannic acid and anhydrous sodium carbonate were of analytical grade, were purchased from Sigma Aldrich (Munich, Germany) and were used for TPC and antioxidant activity analyses. For all HPLC experiments, for mobile phase preparation, ethanol, acetonitrile (ACN) (Riedel-de Haën, Honeywell Riedel-de Haën) and formic acid (Merck, Darmstadt, Germany) were used as received and ultrapure water (Millipore Direct-Q3 UV water purification system equipped with Biopack UF cartridge, Molsheim, France) was used. A Gram-negative bacterium (*E. coli*, strain DH5K) and a Gram-positive bacterium (*B. subtilis*, ATCC 6603) were sourced from the microorganism bank of the Bioreactors Laboratory, Faculty of Chemical Engineering and Biotechnologies, National University of Science and Technology POLITEHNCA Bucharest, and Mueller–Hinton agar and Mueller–Hinton broth (Carl Roth GmbH & Co. KG, Karlsruhe, Germany, 2026) were used for antimicrobial activity.

### 4.2. Preparation of Coffee Samples

Arabica and Robusta green beans were imported from Uganda by Zaniat Com SRL. The Arabica sample (batch no. 103932) was a washed Bugisu AA coffee from the 2025/2026 harvest, grown in eastern Uganda, near the Kenyan border, on the slopes of Mount Elgon, at altitudes ranging from 1200 to 2300 m above sea level. The Arabica sample comprised the SL 14 and SL 24 varieties and received a Specialty Coffee Association score of 80.5. The Robusta sample (batch no. 103996) originated from Central/Eastern Uganda, where it is widely cultivated at altitudes of approximately 900–1500 m above sea level and is predominantly processed using the natural method, which involves sun-drying. Arabica and Robusta green beans were roasted in batches of 120 kg at 202–258 °C (Arabica) and at 221–265 °C (Robusta) for 15 min using a coffee roaster (Giesen W140A Pro, Ulft, The Netherlands) and ground for 6.9 s using an electric grinder (Eureka Atom Specialita 65E, Sesto Fiorentino, Italy). The roasting degree of RCG samples was evaluated using a color meter for coffee roasters (Tonino color meter, Poing, Germany). The RCG samples were subjected to a water-pulsed extraction process at 9 bar and 92–94 °C for 25–30 s using an espresso machine (Astoria Greta, Milan, Italy) corresponding to approximately 9 g of ground coffee used for a beverage preparation. To prevent enzymatic degradation and microbiological spoilage, the remaining SCG was dried using a drying oven (Venticell^®^ 55-ECO line, Gräfelfing, Germany) at 50 °C for 48 h until the dried samples reached a constant weight. The RCG and SCG were stored at −20 °C until analysis.

### 4.3. Soxhlet Extraction from Coffee Samples

The Soxhlet extraction method was used to recover LF and PF from coffee samples. RCG and SCG from Arabica and Robusta varieties of Ugandan origin, were used as coffee matrix types and were subjected to Soxhlet extraction. Two solvents, hexane and ethanol, were selected due to their affinity for lipidic or phenolic compounds. For each extraction experiment, 10 g of RCG/SCG samples and 300 mL of hexane/ethanol were used. In total, 8 independent extraction experiments were conducted for the present study, and each experiment was performed in triplicate. At the end of each extraction experiment, the solvents were removed from the extracts using a rotary evaporator (Hahnvapor, model HS-2000NS, Gimpo, Republic of Korea). The collected coffee extracts were weighed and stored in the freezer at −20 °C until analysis. The results were expressed as extraction yields (g extract/100 g coffee sample).

### 4.4. GC–FID Analysis of Coffee Samples

The contents of glycerides, monoacylglycerides, diacylglycerides, triacylglycerides and free fatty acids in the LFs obtained using hexane extracts were determined by GC-FID using a Bruker Scion 436 gas-chromatograph (Goes, The Netherlands) according to European Standard EN 141052003 and the method described in a previous study [58]. For sample analyses, a ZB-5HT-type fused capillary column with a nitrogen flow of 5 mL/min was used. Because high temperature is necessary to vaporize the glycerides, the injector temperature started at 50 °C and increased at a rate of 50 °C/min until 380 °C with a 2 min hold. The electronic flow controller was set at 2.5 mL/min, and the following oven temperature program was used: 50 °C for 1 min, at a rate of 15 °C/min until 180 °C, at a rate of 7 °C/min until 230 °C and at a rate of 10 °C/min until 370 °C. Detector temperature was set at 380 °C and the gas flows at 15 mL/min for the make-up gas (nitrogen), 15 mL/min for hydrogen, and 300 mL/min for air. The injection volume used was 1 μL. For each analysis, 20 mg of LF was dissolved in 1.5 mL of n-Heptane. The chromatograms of LF samples are illustrated in Figure S1 (Supplementary Material) and glycerides quantification was accomplished based on C18:1 oleic chains reference standards. The results are presented as the percentage of acylglycerides in the oil (g/100 g LF).

### 4.5. ^1^H-NMR Characterization of Coffee Samples

The ^1^H-NMR spectra of the LF obtained using hexane were acquired on a Bruker Avance III HD 600 MHz spectrometer (Bruker, Rheinstetten, Germany), operating at a proton resonance frequency of 600.12 MHz at 25 °C, and equipped with a 5 mm BBI probe with z-gradients. Spectrometer control and spectral processing were performed using TopSpin 3.5.pl6 software. The NMR spectra were recorded using the standard Bruker zg30 pulse sequence, a one-dimensional single-pulse experiment with an acquisition time of 2.73 s, with a 30° excitation pulse, 6 scans, and a relaxation delay of 1 s. For each analysis, 200 mg of LF was dissolved in 0.7 mL of CDCl_3_. The chemical shifts were referenced to the residual CDCl_3_ signal at δ 7.26 ppm. The saturated, mono-unsaturated, and di-unsaturated fatty-acyl proportions were calculated from the normalized ^1^H-NMR signal integrals using the computational procedure reported by Chira et al. [37] and are expressed as molar ratios (mol%) from LF. Weak signals attributed to n-3 fatty-acyl chains that could not be reliably integrated under the acquisition conditions used were interpreted qualitatively and were not assigned quantitative concentrations. The iodine value (IV, g I_2_/100 g LF) and saponification value (SV, mg KOH/g LF) were calculated from the ^1^H-NMR data according to Chira et al. [38] and Ivanova et al. [39], respectively.

### 4.6. Total Phenolic Content of Coffee Samples

The TPC of all eight coffee sample extracts was evaluated with the Folin–Ciocalteu phenol reagent method using four reference phenolic compounds, as gallic acid, caffeic acid, ferulic acid and tannic acid, according to the procedure described in a previous study [59] with some modifications. Briefly, 0.1 g of extract was vortexed at 2400 rpm for 1 min with 10 mL of ethanol. Next, 0.5 mL of ethanolic extract solution was mixed with 2.5 mL of Folin–Ciocalteu solution (10%) for 5 min, and 2 mL of sodium carbonate solution (7.5%) was added. The reaction mixture was incubated in the dark and at room temperature for 60 min, and the absorbance at 765 nm was spectrophotometrically measured. The results were expressed based on reference phenolic compound equivalents such as mg of gallic acid equivalents (GAE)/g extract, mg of caffeic acid equivalents (mg CAE)/g extract, mg of ferulic acid equivalents (mg FAE)/g extract and mg of tannic acid equivalents (mg TAE)/g extract.

### 4.7. HPLC-PDA Analysis of Coffee Samples

The chemical composition of LF obtained using ethanol was determined using the reversed-phase high-performance liquid chromatograph Shimadzu Nexera 2 equipped with a multi-wavelength UV detector (SPD-M30A) (Kyoto, Japan) in the 250–600 nm wavelength domain. The analyses were done at 0.4 mL/min flow using gradient elution with H_2_O/HCOOH (100/2.5) (*v*/*v*)—mobile phase A and acetonitrile/H_2_O/HCOOH (90/10/2.5) (*v*/*v*/*v*)—mobile phase B, using an injection volume of 1 μL for every sample. A more detailed description of the HPLC method was published elsewhere [60]. The samples were filtered through a nylon filter syringe prior to the analyses, and the identification of extract compounds was carried out based on both retention times and spectral similarities and compared with those of standard substances. The results were expressed as mg of phenolic compound/g of extract.

### 4.8. Antioxidant Activity of Coffee Samples

The total antioxidant activities of all eight coffee sample extracts were assessed by the DPPH free radical-scavenging assay with Trolox as a reference antioxidant compound used as a calibration standard according to the procedure described in a previous study [59] with some modifications. Briefly, 0.05 g of extract was vortexed at 2400 rpm for 1 min with 5 mL of ethanol and 5 mL of distilled water. The hydroethanolic solvent (50% *v*/*v*) was used as a control sample. Next, 0.4 mL of hydroethanolic extract solution was mixed with 6 mL of DPPH solution (0.1 mM in ethanol p.a.) and incubated in the dark and at room temperature for 30 min. The absorbance at 517 nm was spectrophotometrically measured (A_control_ is the absorbance of the control sample, and A_sample_ is the absorbance of the extract sample). The results were expressed as the percentage inhibition of free radicals, inhibition % DPPH, determined using Equation (1) presented below.

(1) $$\mathrm{DPPH Inhibition}\;(\%)=\frac{A_{\mathrm{control}}-A_{\mathrm{sample}}}{A_{\mathrm{control}}}\times 100,$$

### 4.9. Antimicrobial Activity of Coffee Samples

To assess the antibacterial properties of all eight coffee samples extracts, a Gram-negative bacterium (*E. coli*) and a Gram-positive bacterium (*B. subtilis*) were used. The antimicrobial tests were performed using a disk diffusion assay. The disks used in antimicrobial experiments had controlled dimensions (a diameter of 6 ± 0.2 mm and a thickness of 0.2 ± 0.05 mm, measured with a Quatros 1–150 mm caliper, Lublin, Poland) and were made from high-purity absorbent filter paper. Their sterilization was performed using a UV lamp (UVGL-58, Multiband UV, UVP, Upland, CA, USA) at 254 nm for 30 min. For bacterial strains, Mueller–Hinton agar was used as the culture medium. The culture medium was sterilized by autoclaving in a Raypa, Catalonia, Spain autoclave at 121 °C for 20 min. The plates were inoculated using a cell depletion technique, with 0.1 mL of cell suspension evenly spread using a Drigalski spatula. Inocula were prepared by transferring colonies of bacterial growth on Mueller–Hinton agar into individual tubes containing 0.85% normal saline solution. The density of the organism suspension was adjusted to a 0.5 McFarland turbidity standard (McFarland Densitometer, Biosan, DEN-1, Riga, Latvia). The volume of extract samples deposited on the discs was 6 µL. Both the discs and the samples were placed aseptically onto the inoculated plates. The plates were incubated (Memmert Incubator IN 110, Büchenbach, Germany) at a specific temperature (37 °C for both strains) for 24 h.

### 4.10. Statistical Analysis

Soxhlet extraction experiments and chemical analysis for the determination of glycerides composition, total phenolic content, antioxidant activity and antimicrobial activity were carried out in triplicate. HPLC-PDA analyses were performed in duplicate. The results were presented as the mean values ± standard deviation (SD). All experimental datasets were subjected to one-way analysis of variance (ANOVA) to determine the variability between the groups and within the groups. Duncan’s multiple range test was applied for post hoc comparisons between experimental values. One ^1^H NMR spectrum was acquired for each of the four LF samples. Accordingly, the reported integral values and NMR-derived parameters represent individual determinations rather than means of replicate measurements. Comparisons among samples are descriptive, and no claims of statistical significance were made on the ^1^H-NMR-derived results.

## 5. Conclusions

This study provides a comprehensive comparative analysis of the antioxidant and antimicrobial properties of liquid and polyphenolic fractions from two matrix types (RCG and SCG) of two varieties of Ugandan coffee (Arabica and Robusta). The extracts obtained from SCG compare favorably with those from RCG in terms of both lipid and polyphenolic compound content. A high-quality LF, namely the coffee oil, (91.82 g triacylglycerides/100 g LF, 49.15 mol% saturated fatty acid chains and 50.15 mol% unsaturated fatty acid chains, 8.98 mg GAE/g LF, 20.91% DPPH inhibition) can be recovered from SCG of the Ugandan Arabica variety with a high extraction yield (10.34 g/100 g SCG). In addition, SCG from both Ugandan Arabica and Robusta varieties are promising resources for the recovery of high-quality PF, namely a polyphenolic extract (12.30 and 7.85 mg GAE/g PF, 0.684 and 0.837 mg/g PF chlorogenic acids, 85.17% and 73.95% DPPH inhibition), with good extraction yields (9.35 and 5.63 g/100 g SCG) and with antimicrobial activity that remains after the coffee brewing step. The coffee oil and polyphenolic extracts can be easily obtained from SCG, and the obtained results in the present study suggest that this waste material is suitable for further valorization. Consequently, these results highlight Ugandan-origin SCG as a promising source of bioactive compounds that can be isolated to assess their applications in specific industries.

## Figures and Tables

**Figure 1:**
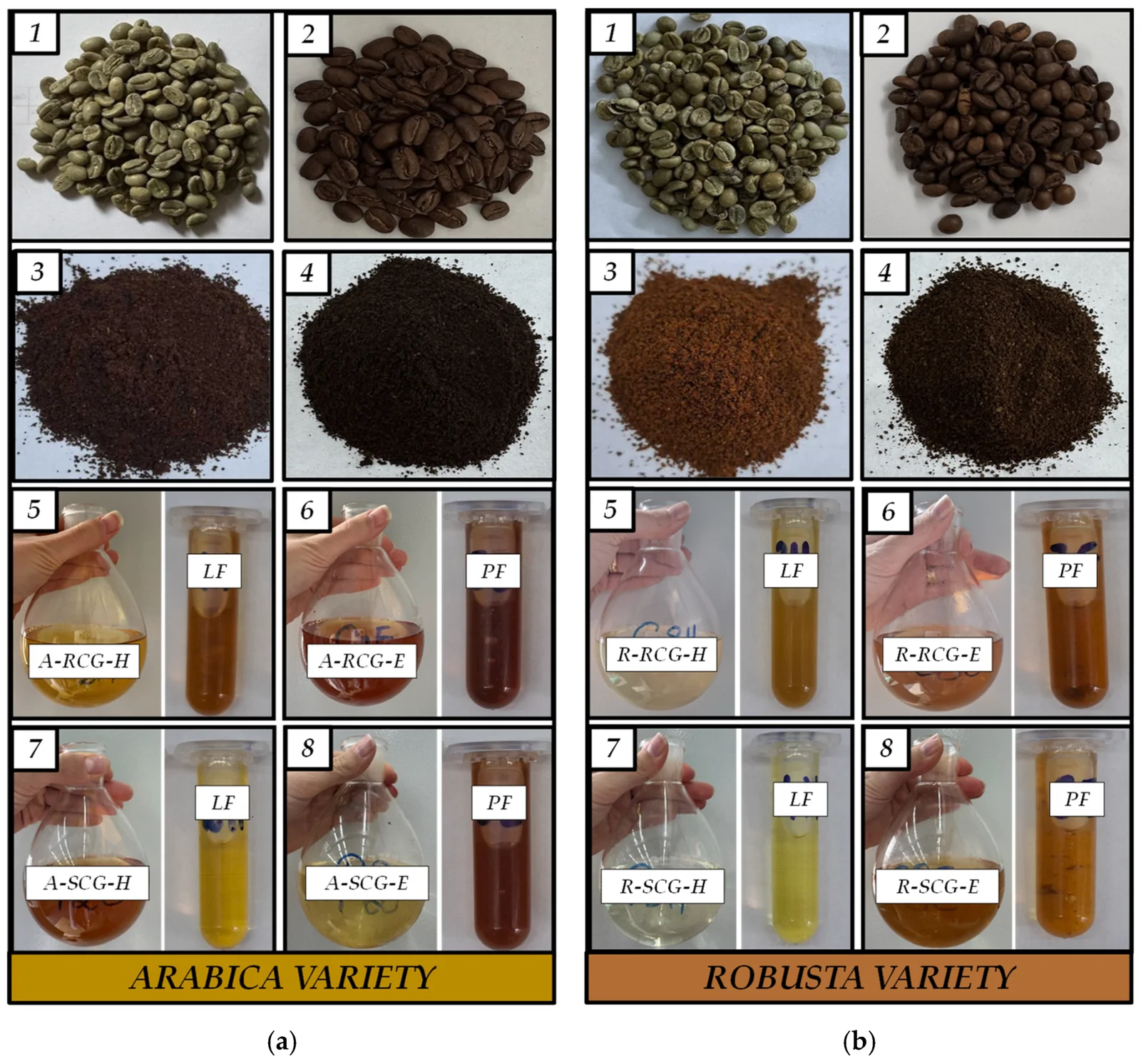
Coffee samples and extracts: 1—green coffee beans, 2—roasted coffee beans, 3—roasted coffee grounds (RCG), 4—spent coffee grounds (SCG), 5—RCG hexane (H) extract and isolated lipid fraction (LF), 6—RCG ethanol (E) extract and isolated polyphenolic fraction (PF), 7—SCG-H extract and isolated LF and 8—SCG-E extract and isolated PF. (**a**) Arabica variety. (**b**) Robusta variety.

**Figure 2:**
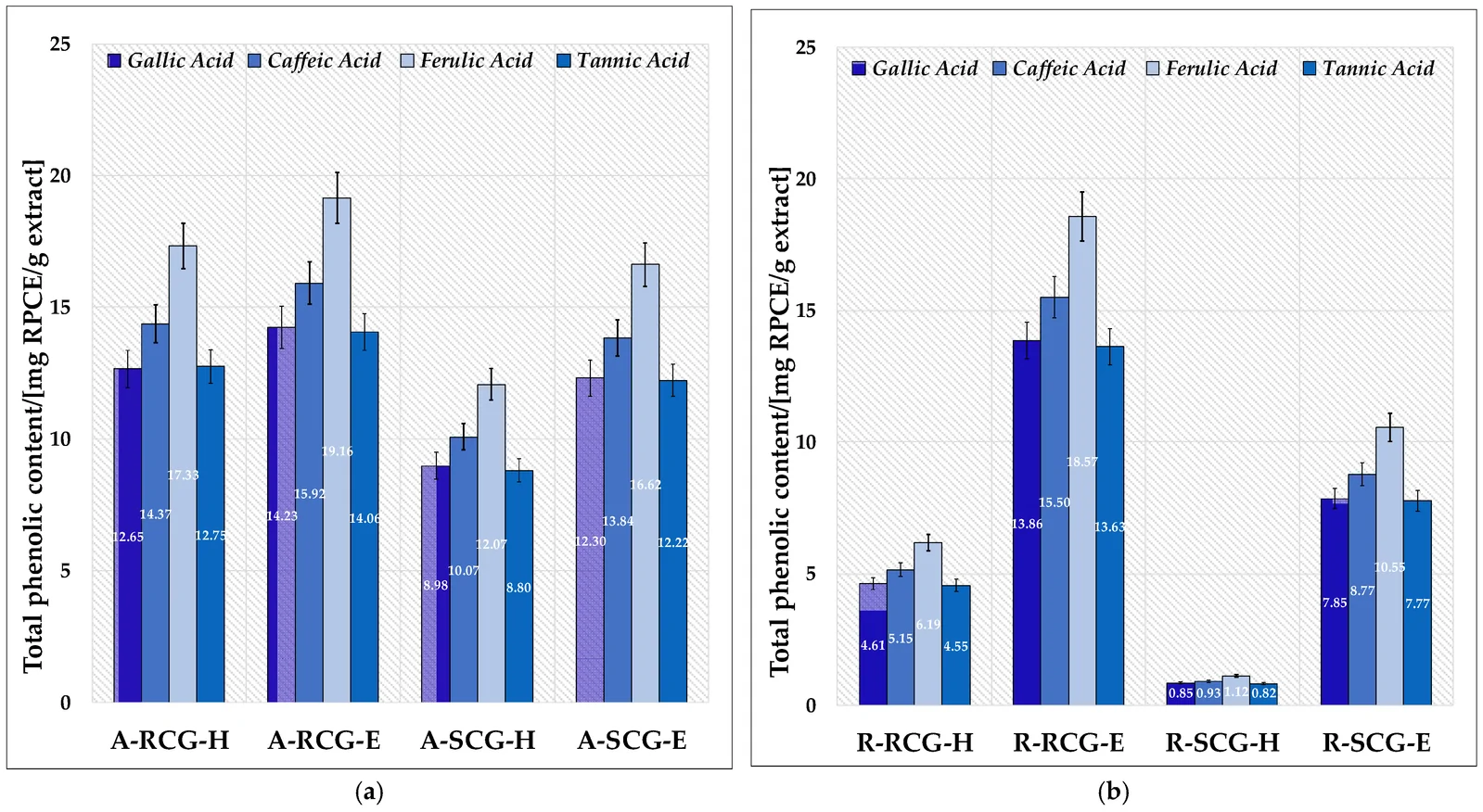
Total phenolic contents (mg reference phenolic compound (RPCE)/mg extract) determined in lipid and polyphenolic fractions of coffee samples of Ugandan origin: (**a**) Arabica variety; (**b**) Robusta variety.

**Figure 3:**
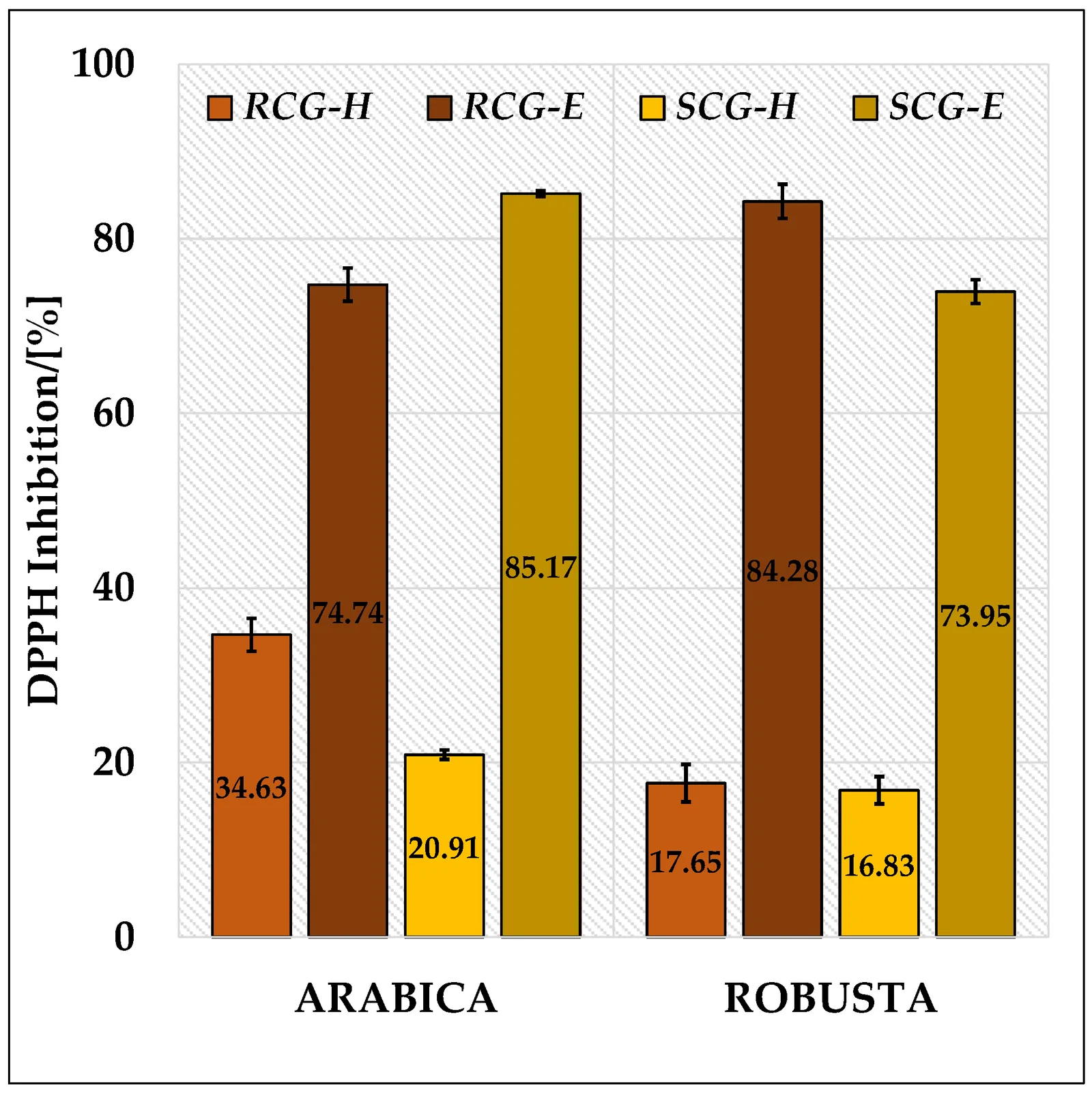
Antioxidant activity (% DPPH inhibition) of lipid and polyphenolic fractions of coffee samples from Arabica and Robusta varieties of Ugandan origin.

**Figure 4:**
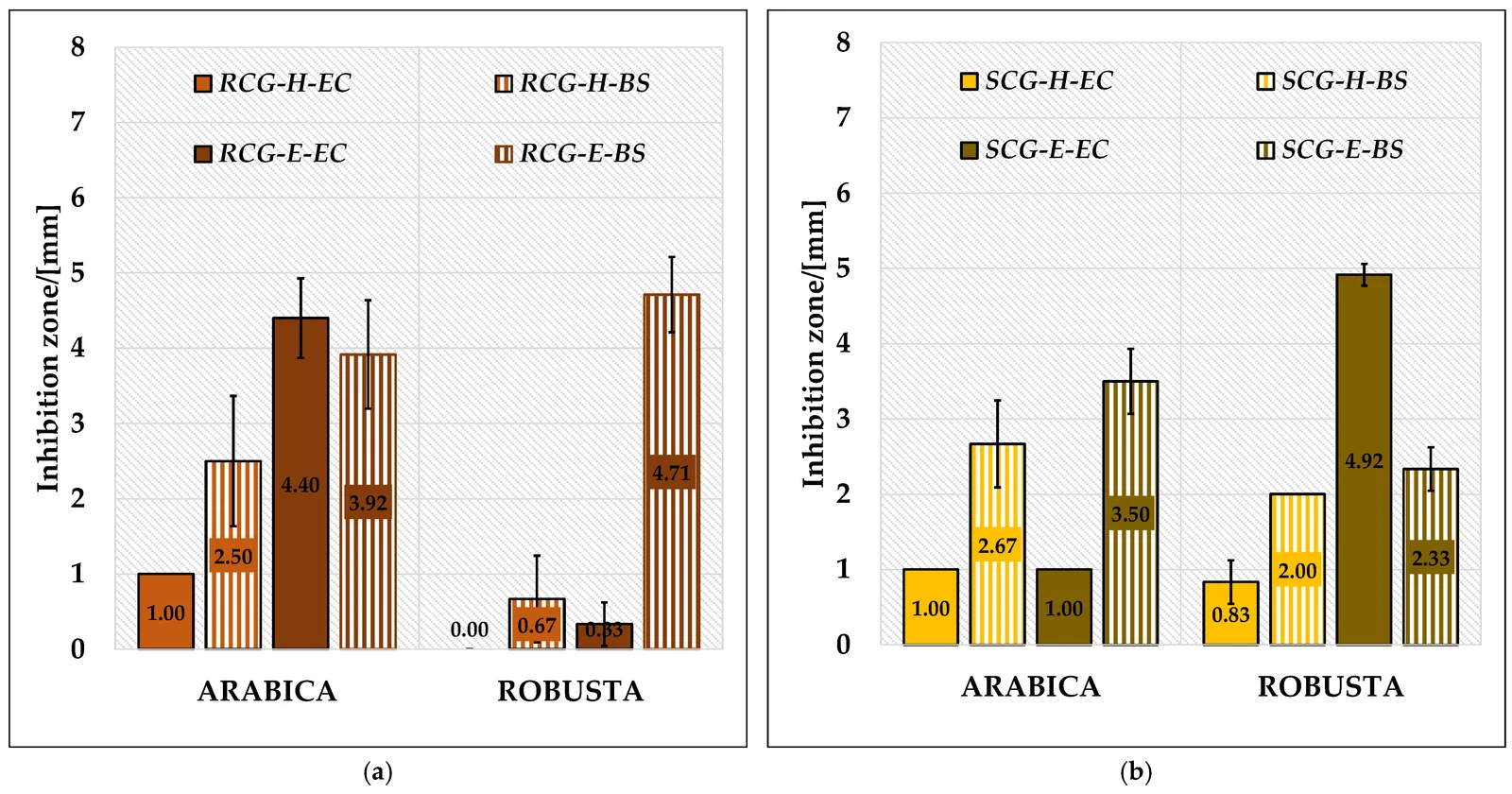
Antimicrobial activity against Gram-negative *Escherichia coli* (EC) and Gram-positive *Bacillus subtilis spizizenii Nakamura* (BS) bacteria, determined for the lipid and polyphenolic fractions of coffee samples from Arabica and Robusta varieties of Ugandan origin: (**a**) RCG; (**b**) SCG.

**Table 1:** Extraction yields (g/100 g coffee sample) for lipid and polyphenolic fractions isolated from Arabica and Robusta coffee varieties of Ugandan origin.

Coffee Variety	Coffee Matrix Type	Extraction Solvent	Fraction Type	Extract ID	Extraction Yield *
Arabica	Roasted Coffee Ground	Hexane	Lipid Fraction	A-RCG-H	11.09 ± 0.49 ^d^
		Ethanol	Polyphenolic Fraction	A-RCG-E	12.74 ± 0.10 ^e^
	Spent Coffee Grounds	Hexane	Lipid Fraction	A-SCG-H	10.34 ± 0.17 ^d^
		Ethanol	Polyphenolic Fraction	A-SCG-E	9.35 ± 0.09 ^c^
Robusta	Roasted Coffee Ground	Hexane	Lipid Fraction	R-RCG-H	6.31 ± 0.08 ^b^
		Ethanol	Polyphenolic Fraction	R-RCG-E	9.30 ± 0.11 ^c^
	Spent Coffee Grounds	Hexane	Lipid Fraction	R-SCG-H	4.09 ± 0.13 ^a^
		Ethanol	Polyphenolic Fraction	R-SCG-E	5.63 ± 0.16 ^b^

* Mean values ± standard deviations (SDs) of three experiments followed by a letter (a–e) indicate that there are no statistically significant differences between extraction yields according to the F test (*p* < 0.05) at the level of significance α = 0.05.

**Table 2:** Acylglycerides profile (g/100 g LF) of lipid fractions of coffee samples from Arabica and Robusta varieties of Ugandan origin.

Extract ID	Free Fatty Acids *	Monoacylglycerides *	Diacylglycerides *	Triacylglycerides *
A-RCG-H	4.80 ± 0.52 ^b^	0.25 ± 0.18 ^a^	3.08 ± 0.49 ^a^	91.86 ± 0.43 ^a^
A-SCG-H	4.74 ± 0.21 ^b^	0.32 ± 0.05 ^b^	3.12 ± 0.25 ^b^	91.82 ± 0.23 ^a^
R-RCG-H	4.21 ± 0.10 ^a^	0.26 ± 0.19 ^a^	3.12 ± 1.87 ^b^	92.41 ± 1.95 ^b^
R-SCG-H	4.36 ± 0.10 ^a^	0.47 ± 0.03 ^c^	3.15 ± 0.27 ^b^	92.01 ± 0.28 ^a^

* Means ± SDs of the three experiments followed by a letter (a–c) indicate that there are no statistically significant differences between glyceride composition according to the F test (*p* < 0.05) at the level of significance α = 0.05.

**Table 3:** ^1^H-NMR characterization of Lipid Fractions of coffee samples from Arabica and Robusta varieties of Ugandan origin.

Extract ID	Fatty-Acyl Composition (mol%) ^&^				PUFA/SFA Ratio	IV ^#^ (g I_2_/100 g LF)	SV ^##^ (mg KOH/g LF)
	Tri- Unsaturated Fatty Acids *	Di- Unsaturated Fatty Acids **	Mono- Unsaturated Fatty Acids ***	Saturated Fatty Acids ****			
A-RCG-H	n.q. ^‡^	44.14	8.90	46.96	0.94	77.07	202.81
A-SCG-H	n.q. ^‡^	38.35	11.80	49.85	0.77	75.24	208.39
R-RCG-H	n.d. ^‡^	34.93	15.64	49.43	0.71	74.03	207.18
R-SCG-H	n.d. ^‡^	31.96	15.31	52.74	0.61	74.23	206.74

^&^ Values were calculated from a single ^1^H NMR spectrum for each sample and represent individual determinations, not means of replicate measurements. Comparisons among samples are descriptive; values were computed using the chemometric equations reported by Chira et al. [37] based on integral values corresponding to ^1^H-NMR resonances presented in Table S2 (Supplementary Material). * Mainly α-linolenate-type chains (C18:3). ** Mainly linoleate-type chains (C18:2). *** Mainly oleate-type chains (C18:1). **** Predominantly palmitate- and stearate-type chains (C16:0 and C18:0); these were not individually resolved by the calculation procedure used. ^#^ Iodine value, computed according to Chira et al. [38]. ^##^ Saponification value, computed according to Ivanova et al., [39]. ^‡^ n.q., not quantified: a weak resonance consistent with n-3 fatty-acyl chains was observed; however, its intensity did not permit reliable quantification under the acquisition conditions used; n.d. = not detected under the acquisition conditions used.

**Table 4:** HPLC-PDA characterization of polyphenolic fractions of coffee samples from Arabica and Robusta varieties of Ugandan origin.

Standard Substance	RT (min) *	Concentration in Polyphenolic Fractions (mg/g PF) **			
		A-RCG-E	A-SCG-E	R-RCG-E	R-SCG-E
gallic acid	3.560 ± 0.007	0.022 ± 0.003	n.d.*	n.d.	n.d.
protocatechuic acid	7.301 ± 0.006	0.017 ± 0.001 ^a^	0.025 ± 0.000 ^b^	0.010 ± 0.000 ^a^	n.d.
neochlorogenic acid	8.457 ± 0.011	0.386 ± 0.002 ^b^	0.318 ± 0.002 ^b^	0.971 ± 0.000 ^c^	0.170 ± 0.001 ^a^
caftaric acid	12.507 ± 0.017	0.040 ± 0.000 ^b^	0.050 ± 0.001 ^c^	0.058 ± 0.001 ^c^	0.011 ± 0.001 ^a^
chlorogenic acid	13.498 ± 0.014	0.776 ± 0.010 ^b^	0.519 ± 0.002 ^a^	2.210 ± 0.000 ^c^	0.514 ± 0.001 ^a^
caffeic acid	15.848 ± 0.015	0.064 ± 0.001 ^c^	0.041 ± 0.000 ^b^	0.048 ± 0.000 ^b^	0.013 ± 0.000 ^a^
(-) epicatechin	17.074 ± 0.000	0.024 ± 0.000	n.d.	n.d.	n.d.
p-coumaric acid	21.994 ± 0.028	0.006 ± 0.000 ^a^	0.009 ± 0.001 ^a^	n.d.	n.d.
trans-ferulic acid	27.094 ± 0.018	0.024 ± 0.000 ^a^	0.032 ± 0.000 ^b^	0.031 ± 0.000 ^b^	0.020 ± 0.000 ^a^
chicoric acid	29.302 ± 0.017	0.150 ± 0.006 ^b^	0.144 ± 0.001 ^b^	0.358 ± 0.001 ^c^	0.080 ± 0.001 ^a^
quercetin	35.440 ± 0.002	n.d.	0.008 ± 0.000	n.d.	n.d.

* RT—retention time, n.d.—not detected. ** Mean values ± standard deviations (SDs) of two experiments followed by a letter (a–c) indicate that there are no statistically significant differences between phenolic compounds according to the F test (*p* < 0.05) at the level of significance α = 0.05.

## Data Availability

Data are available from the corresponding author upon request.

## Supplementary Materials

The following supporting information can be downloaded at: https://www.mdpi.com/article/10.3390/ijms27219906/s1. Reference [36] is cited in the supplementary materials.

## Abbreviations

Following abbreviations are used this manuscript:

RCG	Roasted coffee grounds
SCG	Spent coffee grounds
LF	Lipid fraction
PF	Polyphenolic fraction
