# Supplementary material for: Comparative Study on Coffee and Spent Coffee Grounds of Arabica and Robusta Varieties of Ugandan Origin: Bioactive Properties of Extracts as Circular Resources
Source: Int J Mol Sci. 2026 Nov 5;27(21):9906. doi: 10.3390/ijms27219906 (PMC13763709; doi:10.3390/ijms27219906)
Supplement: Supplementary file 1 [file ijms-27-09906-s001.zip › ijms-4606940-supplementary.pdf]

Comparative Study on Coffee and Spent Coffee Grounds of Arabica and Robusta Varieties of Ugandan Origin: Bioactive Properties of Extracts as Circular Resources

Mihaela Dinu [1], Auras Marius Dumitru [2], Ana Maria Claudia Brezoiu [1], Gabriela Olimpia Isopencu [1,*] and Petrica Iancu [1]

[1] Department of Chemical and Biochemical Engineering, National University of Science and Technology POLITEHNICA Bucharest, 1, Gh. Polizu Street, Building A, Room A056, RO-011061 Bucharest, Romania; mihaela.popescu3010@upb.ro, ana_maria.brezoiu@upb.ro, petrica.iancu@upb.ro

[2] Doctoral School Chemical Engineering and Biotechnologies, National University of Science and Technology POLITEHNICA Bucharest; auras.dumitru@upb.ro

* Correspondence: gabriela.isopencu@upb.ro

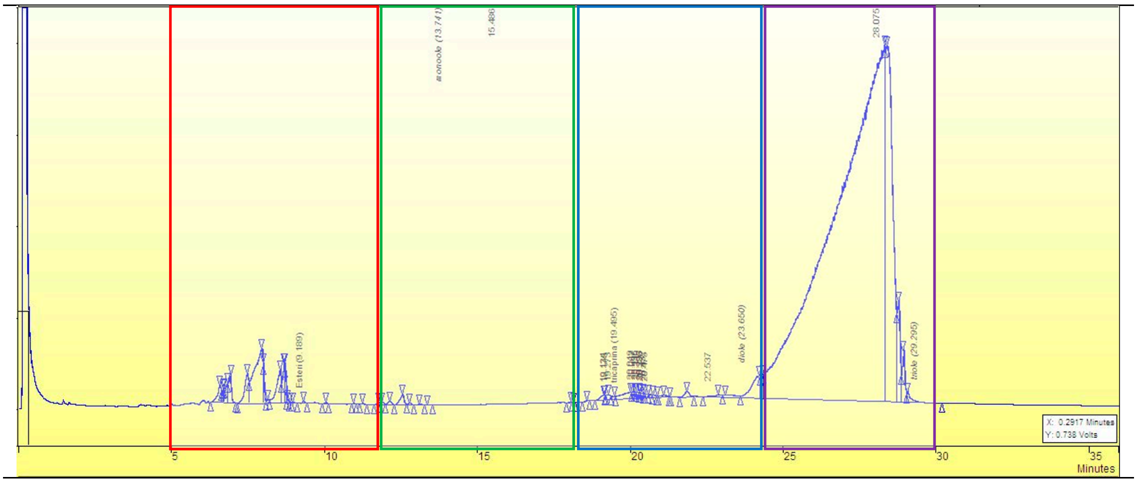

A-RCG-H

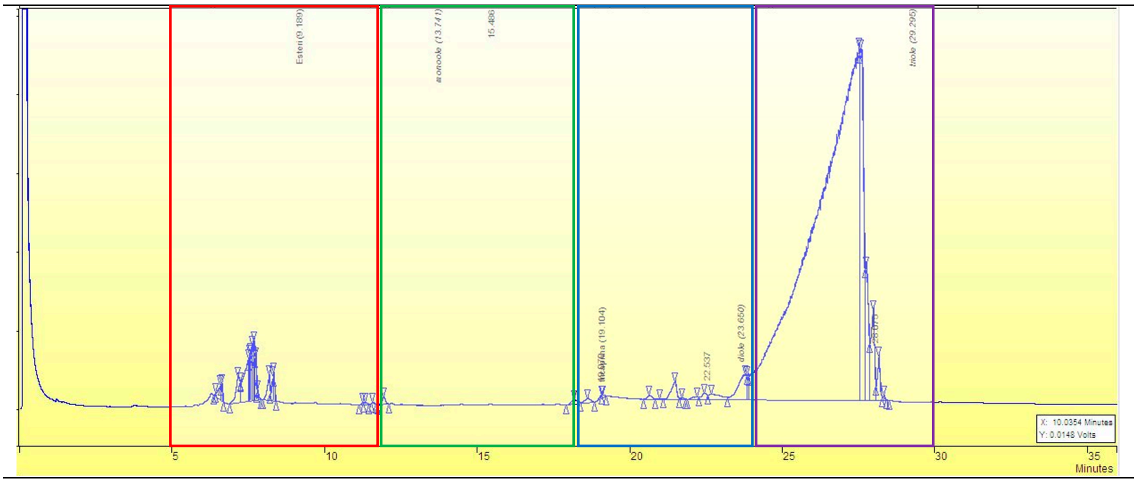

A-SCG-H

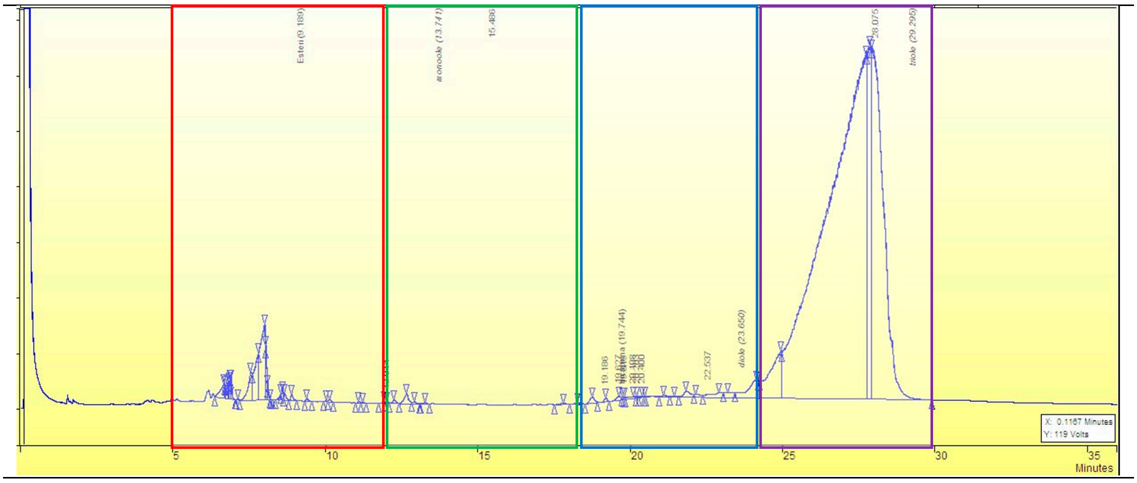

R-RCG-H

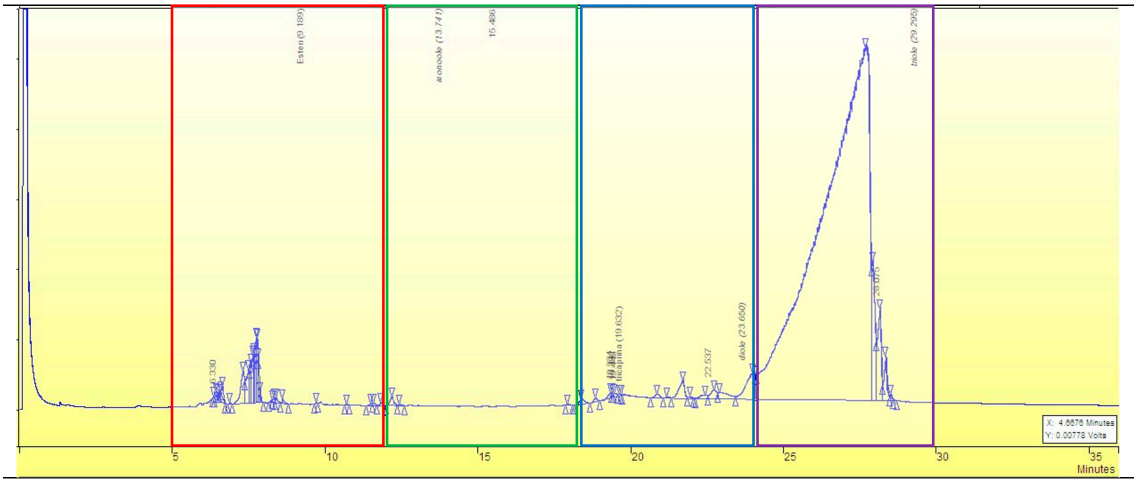

R-SCG-H

Figure S1. GC-FID chromatograms of Lipid Fractions of Arabica and Robusta coffee varieties from Uganda origin.

The acylglycerides detection and quantification using GC-FID method is performed based on the boiling points of the target compounds using four standards, methyl oleate for esters which have close boiling point values to oleic acid, being used as representative for free fatty acids compounds, monolein being used as representative for the monoacylglycerides compounds, diolein being used as representative for the diacylglycerides compounds and triolein being used as representative for triacylglycerides compounds and one internal standard as tricaprin. The detection and quantification of glycerides is performed based on retention times: free fatty acids, the red zone between 5-12 min, monoacylglycerides, the green zone between 12-18 min, diacylglycerides, the blue zone between 18-24 min) and triacylglycerides the purple zone between 24-30 min. The peak areas generated within each color-coded retention time window are integrated. They are then calibrated against the response factors of their respective single-acid standards (all based on C18:1 oleic chains) to calculate the concentration of each lipid class in the sample.

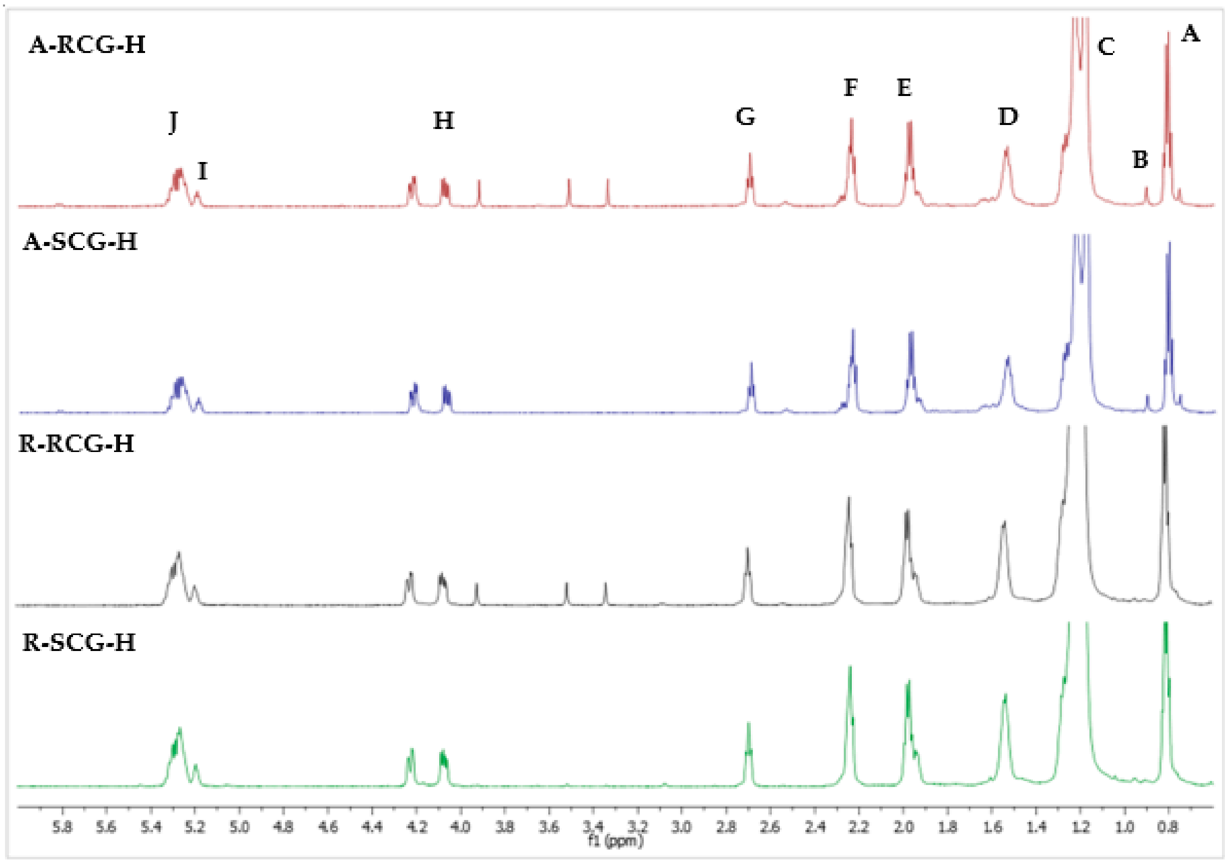

Figure S2. Stacked ^{1}H-NMR spectra of Lipid Fractions of coffee samples from Arabica and Robusta varieties from Uganda origin. Peak assignments correspond to Table S1.

Table S1. Chemical shifts and peak assignment of ^{1}H-NMR spectra of fats and oils [1]

Signal	δ (ppm)	Proton	Compound
A	0.96	-CH=CH-CH$_2$-C*H*$_3$	linolenic acid
B	0.85	-CH$_2$-CH$_2$-CH$_2$-C*H*$_3$	all acids except butyric acid and linolenic acid
C	1.24	-(C*H*$_2$)$_n$-	all fatty acids
D	1.64	-C*H*$_2$-CH$_2$-COO-	all fatty acids
E	2.02	-CH2-CH=CH-	all unsaturated fatty acids
F	2.26	-C*H*$_2$-COO-	all fatty acids
G	2.76	-CH=CH-C*H*$_2$-CH=CH-	n-6 (linoleic) acid and n-3 (linolenic) acid
H	4.19	-C*H*$_2$OCOR	H in the *sn*-1/3 position of the glycerol backbone
I	5.15	-C*H*OCOR	H in the *sn*-2 position of the glycerol backbone

Signal assignments followed Knothe and Kenar [1] and are presented in Table S1. The integrated signals comprised the combined terminal methyl resonances at approximately δ 0.85 and 0.96 ppm (A+B); the methylene resonances at δ 1.24 ppm (C), 1.64 ppm (D), 2.02 ppm (E, allylic), 2.26 ppm (F, α to the carbonyl group), and 2.76 ppm (G, bis-allylic); the glycerol methylene resonance at approximately δ 4.19 ppm (H); and the combined glycerol methine and olefinic resonances at approximately δ 5.15 and 5.29 ppm (I+J). Integral values were normalized by setting the combined terminal methyl signal area, I_{A+B}, to 3.0000 for each sample. The normalized integral values corresponding to the spectra shown in Figure S2 are provided in Table S2.

Table S2. Normalized integrated signal areas corresponding to the ^{1}H NMR spectra of Lipid Fractions of coffee samples from Arabica and Robusta varieties from Uganda origin shown in Figure S2.

	^{1}H-NMR integral values*							
**Extract ID**	I_{A+B}	I_C	I_D	I_E	I_F	I_G	I_H	I_{I+J}
**A-RCG-H**	3.0000	18.4264	2.6672	2.1215	1.9199	0.8828	1.2215	1.9839
**A-SCG-H**	3.0000	17.7657	2.6477	2.0060	1.7597	0.7670	1.1556	1.8763
**R-RCG-H**	3.0000	17.7689	2.2903	2.0229	1.7471	0.6986	1.1091	1.8185
**R-SCG-H**	3.0000	17.0594	2.2399	1.8905	1.6438	0.6391	1.0610	1.7643

*integral values are expressed in relative units and were normalized by setting I_{A+B} (the combined area of the terminal methyl signals A and B) to 3.0000 for each sample. Signal labels correspond to those used in the peak-assignment table. I_{A+B} and I_{I+J} denote the combined integrated areas of signals A and B, and I and J, respectively.

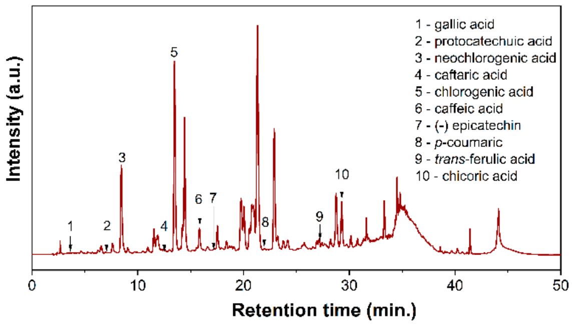

A-RCG-E

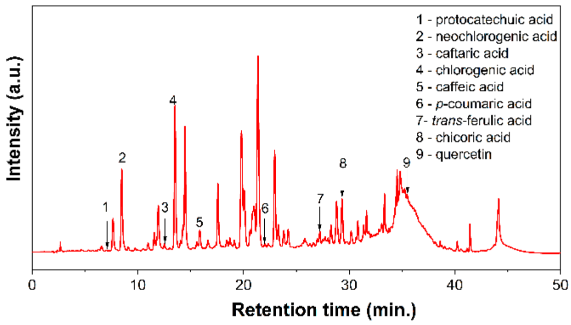

A-SCG-E

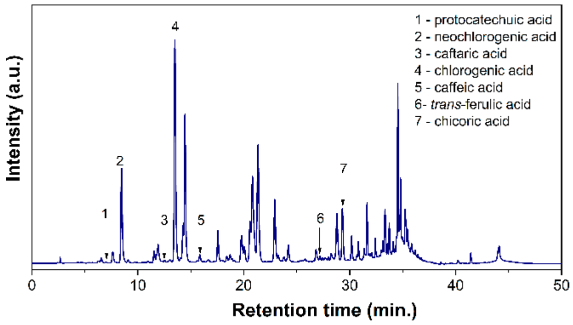

R-RCG-E

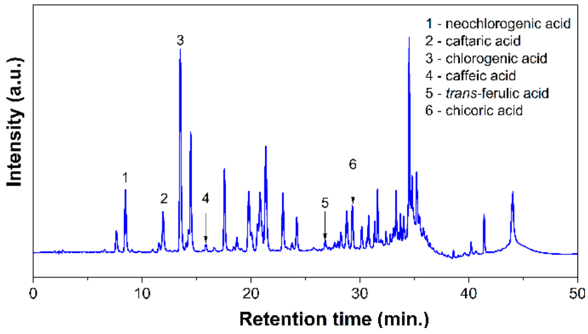

R-SCG-E

Figure S3. Chromatograms of Polyphenolic Fractions of coffee samples from Arabica and Robusta varieties from Uganda origin recorded at 326 nm.

Standard HPLC-grade substances used for chromatographic analyses were purchased and used as received from Sigma/ Sigma-Aldrich (Merck Group): caffeic acid, catechin hydrate, rosmarinic acid and neochlorogenic acid (98 %, HPLC), trans-ferulic acid (>98 %, GC), trans-resveratrol (certified reference material), p-coumaric acid and delphinidin chloride (analytical standard), rutin hydrate (94 %, HPLC), myricetin (>96 %, HPLC), kaempferol (>97 %), malvidin chloride, cyanidin chloride and quercetin (>95 %, HPLC), pelargonidin chloride (90 %, HPLC), malvidin-3-galactoside (analytical standard); from Carl-Roth: peonidin chloride (HPLC), from Alfa Aesar: gallic acid (98 %), from TCI: chicoric acid (>98 %), (−) epicatechin, ellagic acid dihydrate and protocatechuic acid (>98 %, HPLC), vanillic acid and vanillin (>98 %, GC), from Molekula GmbH: caftaric acid (analytical standard) and syringic acid (>98.5 %) and from HWI group: chlorogenic acid (primary references standard).

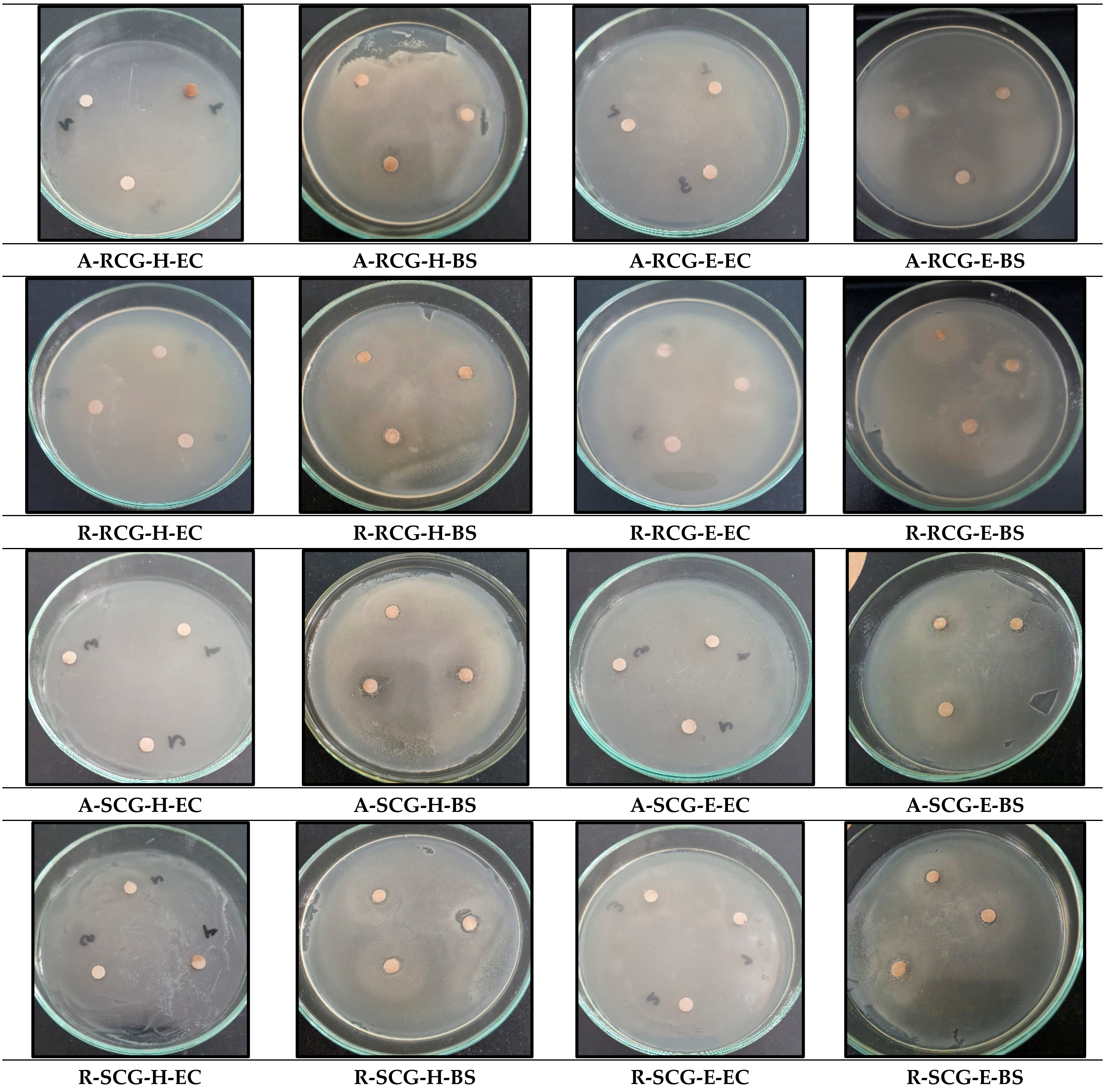

Figure S4. Antimicrobial activity against Gram-negative *Escherichia coli* (EC) and Gram - positive *Bacillus subtilis spizizenii Nakamura* (BS) bacteria illustrated for the Lipid and Polyphenolic Fractions of coffee samples from Arabica and Robusta varieties from Uganda origin.

References

1. Knothe, G.; Kenar, J. A. Determination of the fatty acid profile by 1H-NMR Spectroscopy. *Eur. J Lipid Sci. Technol.* **2024**, 106, 88–96.
